# Conference Report: The ESF Programme on Integrated Approaches for Functional Genomics. Workshop on ‘Data Integration in Functional Genomics and Proteomics’

**DOI:** 10.1002/cfg.134

**Published:** 2002-02

**Authors:** Pierre-Alain Binz, Annette Martin, Mike Taussig, Antoine de Daruvar

**Affiliations:** 1 Swiss Institute of Bioinformatics Proteome Informatics Group 1 Rue Michel Servet Geneve 4 CH-1211 Switzerland; 2 The Babraham Institute Cambridge UK; 3 LION bioscience Bordeaux France


**Comparative and Functional Genomics**
*Comp Funct Genom* 2002; **3**: 16–21.
**DOI:** 10.1002/cfg.134

**Feature**

### Conference Report: The ESF Programme on Integrated Approaches for Functional Genomics. Workshop on 'Data Integration in Functional Genomics and Proteomics'

October 15–17th, 2001; Swiss Institute of Bioinformatics, Geneva, Switzerland

Pierre-Alain Binz[1]*, Annette Martin[2], Mike Taussig[2] and Antoine de Daruvar[3]

[1] *Swiss Institute of Bioinformatics, Switzerland*
[2] *The Babraham Institute, Cambridge, UK*
[3] *LION bioscience, Bordeaux, France*

**Correspondence to:*
*Swiss Institute of Bioinformatics,*
*Proteome Informatics Group,*
*1 Rue Michel Servet, CH-1211*
*Geneve 4, Switzerland.*
*E-mail:*
*Pierre-Alain.Binz@isb-sib.ch*

Published online:
2 January 2002

### Introduction

Beyond an analysis of gene function at the genome level, the ultimate goal of functional genomics is to understand the organisation and coordinated operation of the cell. Integration of data and information is an essential feature of all the steps leading from the production of experimental results to the modelling of a complete cell. The Geneva workshop, organised within the framework of the European Science Foundation (ESF) Programme on Integrated Approaches for Functional Genomics (**http://www.functionalgenomics.org.uk**), provided an excellent opportunity to review the issue of data integration from various perspectives. It brought together scientists with different backgrounds (biologists, bioinformaticians), who currently participate in consortia involving or requiring the integration of heterogeneous biological data. The workshop dedicated equal time to presentations and discussions in order to optimise knowledge distribution and sharing.

The first session was devoted to existing functional genomics projects, where diverse sets of experimental approaches are applied to a model organism or given project. For these, both the scientific objectives and data management strategies were described. Another set of presentations focused on 'data integration requirements' in proteomics. In this rapidly evolving area, data integration is a key issue for technological developments that are being carried out in both academic and industrial contexts. Data integration approaches were then presented by scientists involved in the design and maintenance of public database resources. The last session was devoted to bioinformatics and gave an overview of state of the art approaches for building up information systems with good capabilities with respect to data integration. The workshop concluded with an open discussion on bottlenecks and perspectives that emerged from the presentations.

As a number of speakers have submitted reviews based on their presentations that are appearing in this issue of CFG and in the next one, in this report

Copyright © 2002 John Wiley & Sons, Ltd.

we only briefly mention the content of their contributions at the workshop.

### Sessions and subsequent discussions

### European functional genomics projects: scientific issues and status

**Uwe Kaerst** (**Gesellschaft für Biotechnologische Forschung, Braunschweig, Germany**) introduced the European Union funded REALIS project (Molecular strategies for adaptation and survival: Post-genomic analysis of the lifestyles of *Listeria monocytogenes* (LM) in the environment and the infected host) which commenced in February 2000 (see Kaerst *et al.*, in this issue). As the LM genome is already known, the project aims to completely decipher all the genes required for survival and adaptation of LM within an infected host and for responses to the external environment. Using genomic and post-genomic tools, REALIS also seeks to precisely address questions regarding the evolutionary relationships between pathogenic and non-pathogenic *Listeria* and to define the qualities of particularly successful clonal pathovariants which cause disease. Workpackages are focused on transcriptomics, proteomics, large-scale generation of mutants, central regulon analysis, comparative genomics and bioinformatics. The latter workpackage aims to develop an integrated bioinformatics database, based on the SRS6 system.

**Peter Rice** (**LION bioscience, UK**) described RIBDB, an integrated database of *Listeria* experimental data. Based on SRS6 (**http://srs.ebi.ac.uk**), it is hosted on a central website and acts as the REALIS consortium support for internal queries. It is currently populated with public database sequences of *Listeria*, gene lists, annotations for *L. monocytogenes*, expression data and proteomic spot annotations (see Rice *et al.*, in this issue).

**Philippe Glaser** (**Institut Pasteur, Paris, France**) presented the comparative genomics activity that will be implemented as part of the REALIS project. He depicted phylogenetic relationships between various *Listeria spp.*, *Bacillus subtilis* and *Mycobacterium genitalium*. Symmetry, similarities and structure conservation were discussed, based on analysis of genomic sequences.

**Javier Paz Ares** (**Centro Nacional de Biotecnologia, Madrid, Spain**) presented the aims and structures of the REGIA (Regulatory Gene Initiative in *Arabidopsis*) project (see the review in our next issue), which started in February 2000 with two years of funding. It is mainly focused on the characterisation of transcription factors (TFs) and their relevance to plant breeding programs. The seven workpackages include expression patterns, identification of mutant and TF loci, ectopic expression, phenotype analysis using high throughput metabolic profiling, interaction using two-hybrid screening (Y2H) and bioinformatics. Proteomics is not included in this project. About 1500 TFs are recognised, from which, about 420 zinc finger proteins have thus far been predicted.

The information storage, analysis and web representation of REGIA (see the review in our next issue)were presented by **Alfonso Valencia** (**Centro Nacional de Biotecnologia, Madrid, Spain**). About 30 groups interact with the database. The architecture uses XML to transfer the data to the central relational database. No raw data are introduced. While the metabolic data and Y2H data are formalised and normalised, there is no such standard for transcriptional and mutant analyses. There is a fundamental difficulty in homogenising the experimental and analytical procedures, when various groups do not employ the same approaches. This is yet to be completely resolved.

**Wilhelm Stiekema** (**Wageningen University, The Netherlands**) presented the goals of the recently funded EU PLANET (European Plant Genome Database Network). With 8 partner groups, the workpackages include topics such as ontology, high-density maps, data mining tools, haplomaps, diversity maps, metabolome profiling, genomics, and proteomics. The study involves analysis of gene function for plant protection. Much of the available data, partially that coming from Germplasm DB (6 million accessions, including molecular genetic fingerprints of more than 250 markers), will be combined with data generated by the consortium to build an annotation pipeline. This will probably be based on flat files and automated using Perl scripts. He also mentioned EXOTIC (the Exon Trapping Insert Consortium), the aim of which is to detect the spatial and temporal expression patterns of approximately 5000 *Arabidopsis* genes, and to identify and characterise their regulatory elements.

**Colin Harwood** (**Newcastle University, UK**) presented the activities of BACELL (From gene regulation to gene function: regulatory networks in

Copyright © 2002 John Wiley & Sons, Ltd.

the model Gram-positive bacterium *Bacillus subtilis*, see Harwood and Moszer in this issue). The 11 groups forming this EU funded consortium have divided their activities into four workpackages. Transcriptomic and proteomic approaches are used to observe responses to environmental changes (stimulons and regulons) and to characterise regulatory proteins. Global regulatory networks will be built using data integration and existing databases, such as MICADO (**http://locus.jouy.inra.fr/cgi-bin/genmic/madbase/progs/madbase.operl/**) and Sub2D (**http://microbio2.biologie.uni-greifswald.de : 8880/**).

**Peter Jungblut** (**Max-Planck-Institute for Infection Biology, Berlin, Germany**) discussed the EBP thematic network (Comparative analysis of proteome modulation in human pathogenic bacteria for the identification of new vaccines, diagnostics and antibacterial drug targets). It comprises 10 workpackages including studies on 6 pathogenic bacteria. Besides its functional goals, the network aims to build a database system, incorporating mainly proteomic but also transcriptomic data (see Pleissner *et al.*, in this issue). Two complementary alternative approaches have been proposed to the consortium members: a centralised database in Berlin (see Pleissner *et al.*, in our next issue) and a system based on the federated model of World-2DPAGE (**http://www.expasy.org/ch2d/2d-index.html**).

**Clive Edwards** (**University of Liverpool, UK**) introduced a field not frequently considered in standard approaches to organism study, namely molecular ecology. He highlighted the importance of monitoring active prokaryotes in their natural environments, as these organisms are involved in key biogeochemical cycles and may have pathogenic incidences. In the last 10 to 15 years, molecular biology methods and particularly sequencing of 16S rRNA have been used to detect "functional genes" in prokaryotes. The discovery of such genes does not necessarily reflect actual activity, however. DNA array technology could provide a possible alternative for these approaches. One major technical difficulty is that of culturing the vast majority of marine prokaryotes. Proteomic approaches are proposed in order to identify active prokaryotes and environmentally induced active protein synthesis, using two-dimensional polyacrylamide gel electrophoresis (2-D PAGE) coupled with pulse-labelling experiments and mass spectrometry (MS). The cellular origin of these proteins could be observed via fluorescently labelled probes.

### Session on proteomics

**Manfredo Quadroni** (**University of Lausanne, Switzerland**) addressed the proteomics issues of goals, technologies and quality requirements. While defining the first phase of proteomics analysis as the production of experimental data, he discussed the difficulty of capturing and comparing 2-D PAGE and MS results when there exists such a degree of protocol heterogeneity between different labs. The second obstacle corresponds to data handling and analysis. He pointed out some requirements related to data management, database usefulness and data quality assessment. Here there is a need for detailed descriptions of sample origin and preparation, relationship to expression levels, subcellular localisation, 3-D structure, biological activity, description of post-translational modifications and interpretation of protein interaction maps, among others. He also discussed the quality difference between curated and archived databases.

**Ron Appel** (**Swiss Institute of Bioinformatics, Geneva, Switzerland**) focused on quality issues applied to 2-D PAGE image analysis and related databases. At the user level, efforts have to be made to describe all parameters describing the generation of 2-D PAGE images. These include sample choice and preparation, the protocols for the 2-D PAGE separation, the gel staining and digitising steps. The image analysis software should, on its side, deliver information on the type of algorithm used for spot detection and matching, together with associated parameters and a measure of confidence. The principle 'garbage in, garbage out' can be therefore understood as 'quality in, quality out'. He presented an application of, and the related challenges for, the Melanie image analysis software (**http://www.expasy.org/melanie**), and the advantages and issues of the Federated Model of 2-D PAGE databases.

**Joel Vandekerckhove** (**University of Ghent, Belgium**) presented alternatives to 2-D PAGE based protein identification methodologies. Besides the 'multi-dimension liquid separation protein identification technique' (MudPIT) and the ICAT technique, he introduced a new methodology involving specific amino acid labelling and diagonal chromatography. He illustrated the advantages of this technique, compared to 2-D PAGE based identification, in enhancing sensitivity.

**Denis Hochstrasser** (**Geneva University, Geneva University Hospital, GeneProt, Switzerland**) introduced the principle and the capacities of the

Copyright © 2002 John Wiley & Sons, Ltd. 

molecular scanner, a parallel, high-throughput method of analysing 2-D PAGE separated protein samples. He then presented the industrial approach that GeneProt (**http://www.geneprot.com**) has chosen to analyse entire proteomes with high sensitivity.

### Session on databases

**Amos Bairoch** (**Swiss Institute of Bioinformatics, Geneva, Switzerland**) presented the current status of the SWISS-PROT knowledge base (**http://www.expasy.org/sprot**) and related databases. As the integrated data comes from various types of sources, he pointed out that the maintenance of the information and of the cross-references to external databases is difficult. In the case of cross-references to Medline/PubMed, current stability is good. However, it is difficult to link directly to an article as the journals use different systems. SWISS-PROT has cross-links to 46 databases and 21 extra created on the fly. There are links to 38 2D-PAGE databases on the web that are providing annotation for about 200 images. However there are currently no public MS databases available on the web. The DR lines (Cross-references) are in general difficult to maintain, as various databases have unstable unique identifiers, which necessitate manual curation. The CC database is used to link information resources relevant to only a small number of proteins. These types of annotation are particularly labour intensive. A new feature is the OX line, which links to the NCBI taxonomy. As taxonomy frequently changes, these lines have to be updated frequently, almost every week. In order to help annotation, controlled vocabularies are used for several topics, including keywords, journal abbreviations, tissue, plasmid name, catalytic activity, etc. At the sequence level, up to 12 000 variants have been validated and annotated. There are also links to other information, including synonyms, MS data, cofactors, and pathways. In the future, SWISS-PROT will also be distributed in XML.

**Henning Hermjakob** (**European Bioinformatics Institute, Hinxton, UK**) introduced present and future projects of the European Bioinformatics Institute (EBI, **http://www.ebi.ac.uk**). Temblor, accepted in May 2001 for three years, is a 25-member consortium (see Hermjakob and Apweiler, in this issue). It will provide a highly integrated view of genomic and proteomic data (Integr8) by drawing on databases maintained at major bioinformatics centres in Europe, and by creating new and important resources for protein-protein interactions (IntAct), structural (EMSD) and microarray (DESPRAD) data.

**Ivan Moszer** (**Institut Pasteur, Paris, France**) presented the Subtilist DB (**http://genolist.pasteur.fr/SubtiList/**), dedicated to *Bacillus subtilis*. Historically based on the MICADO and Sub2D databases, the main features of Subtilist include enhanced and verified information on contigs, genes and proteins, EMBL entries and bibliographic references. As well as sequence correction, applied to more than 200 genes, a significant number of ‘unknown’ genes have been labelled and associated with functions. Subtilist is currently being adapted to handle transcriptomics data that are produced in the framework of the BACELL project (above). The database design is done very carefully in order to store all the information, which will be useful to support quality control and expression data analysis. The system can be extended with additional tools and links and can also be generalised for use with other organisms.

### Session on bioinformatics

**Robert Stevens** (**Manchester University, UK**) presented a talk on ontologies, a field which is increasingly used within the world of genomics and functional genomics. He presented the components of an ontology and the process of building one in order to capture knowledge about a domain (see Roberts, in this issue).

**Paul Van der Vet** (**University of Twente, Netherlands**) talked about C2M, a configurable chemical middleware. The current difficulty experienced in exchanging information from heterogeneous resources is related to format multiplicity. Ideally, users should have several tools at their disposal, such as a wrapper (data converter) generator that uses high-level format description, a code generator that turns these descriptions into an appropriate program code, a compiler, and a documentor that turns these descriptions into a human readable documentation. C2M is a prototype that aims to meet these required specifications (see Van der Vet, in issue 2/6).

**Anne Morgat** (**INRIA, Montbonnot-Saint Martin, France**) introduced Panoramix, a two-year project aimed at federating a set of knowledge bases dedicated to microbial genome annotation (see the paper in our next issue). From 49 known microbial

Copyright © 2002 John Wiley & Sons, Ltd.

genomes, two have been chosen as reference organisms. Four activities are linked to Panoramix: Genomix (genomic information from genome sequences), Proteix (protein information, based on extraction from SWISS-PROT), Organix (organic compounds involved in biological processes) and Metabolix, (dealing with the description of metabolic pathways). Metabolix faces challenges associated with heterogeneous user points of view (eg., chemists see chemical compounds, biochemists see enzymes, geneticists see genes encoding proteins, computer scientists see graphs encoding reactions). Anne highlighted the difficulty of integrating inconsistencies and incomplete data with existing generalised and specialised databases. An example is the comparison of chemical compound names as CAS numbers and/or structural representations.

**Denis Shields** (**Royal College of Surgeons, Ireland**) presented an approach for integrating genotyping data. He talked about the EU prospective cardiology project, MORGAM, and the World Health Organisation's MONICA project (**http://www.ktl.fi/monica/index.html**). The idea of the bioinformatics activity here is to integrate genetic variation information coming from phenotypic, metabolic, proteomic, transcriptomic and genetic data (see Shields and O'Halloran, in this issue). The principle of the planned architecture is to use a Laboratory Information Management System (LIMS) to handle raw data and to centralise them in a database for later analysis using statistical and data mining tools. The design of the centralised database is still not finalised and will depend on the types of data generated.

### Bottlenecks and perspectives

The last session took the format of an open discussion based on a number of predefined topics.

**The possibility of sharing data and database schema**. There is an obvious need to compare and perhaps exchange database schema between ongoing projects. As presented here, most of the projects involve creation of a database and all developers had encountered the difficulty of heterogeneity of format and data description in existing databases. The discussion highlighted the practical difficulties of sharing database schema from different networks, owing to several reasons. Some databases are not at a stage suitable for sharing information or are not stable yet. In addition, there are intellectual property issues and restrictions from contracted partners (commercial or otherwise). At this point it was noted that the EU has only recently started to fund bioinformatics activity in most of these projects. It was suggested that public funding ought to be related to public access to the information in the databases and their schema. This is particularly important as numerous publications are based on data that are not publicly available.

**Post-project maintenance of the databases** is also required, which needs additional funding. Even if funding of sequence databases, such as SWISS-PROT or EMBL, is more or less stable today, this is not the case for the newer types of functional genomics related databases. There is currently no source of EU funding for curating these highly important data. Currently, at the end of the granted period, there is no rule and no funding assigned to the maintenance of the data collected, hence posing a considerable threat that all of this valuable data may be lost. This topic has to be further discussed and practical solutions have to be reached.

A consensus was agreed amongst the projects that have been running for more than one year that **interaction between biologists and bioinformaticians** has to be improved. The current lack of interaction relates to the difficulty bioinformaticians face in predicting the needs of biologists and evaluating the future possibilities of this rapidly evolving technology. The current tactic uses a pragmatic approach of matching a 'prototype' model. This then requires specific redesigning and optimisation. It is only recently that the MGET and EU PLANET projects have been able to define a workpackage specific to dealing with this issue. Clearly, biologists need to understand the capabilities of bioinformatics to extract and interpret the masses of data that are generated by their research to assist them further (see Van der Vet *et al.*, in issue 2/6).

**Status of modelling and systems biology.** A new and far-reaching area, this topic was generally not represented in the various projects here. The issue pertains to the collection of large amounts of data from extraneous sources, which is not always possible within these projects. In addition, the lack of common crossover between databases is currently a limiting factor. A proposal has been submitted for an ESF programme workshop in 2002, focusing on this issue. The same arguments are true when initiating **data mining** activities; here, a workshop may be scheduled for 2003.

Copyright © 2002 John Wiley & Sons, Ltd. 

### Conclusions and future

The workshop, as an exploratory experience, was considered very interesting and successful by a vast majority of the participants. The broad range of individual expertise raised interesting issues and highlighted the need for multidisciplinary crossover between the partners of EU functional genomics projects. This workshop was aimed at assembling combined knowledge about the current status of these European projects, which was a unique opportunity. In addition to the comments made above, some further proposals have been made for future activities and improvements to the format of workshops such as this. Now that an initial status on the various projects has been assessed, further shorter workshops focusing on more specific topics should be organized, perhaps as satellites of conferences, in order to maintain the momentum of sharing and integration begun here. The current ESF programme may finance several training courses or personnel exchanges, within the programme areas, in order to facilitate crossover and integration of bioinformatics and biology. Efforts are still being made to facilitate and enhance the content (quality and format) of databases and to provide the end users with powerful and transparent, optimized tools. This may be made possible by encouraging better communication between established and future projects.

### Acknowledgement

REALIS, REGIA, BACELL, EBP are funded under the Fifth Framework Programme by the Quality of Life and Management of Living Resources Programme (**http://europa.eu.int/comm/research/quality-of-life.html**) of the European Commission. Description of the projects can be found on the Community R&D Information Service (CORDIS) server (**http://www.cordis.lu/**).

The organisers would like to thank the ESF for financing the workshop and Geneva Bioinformatics, GeneProt and LION bioscience for generous sponsorship. We are also grateful to the Medical Faculty of the Geneva University, which provided the conference rooms and infrastructure. Thanks also go to Vanessa Lenglart who entertained the participants with a piano recital in Cartigny, a charming village in the Geneva countryside.

Copyright © 2002 John Wiley & Sons, Ltd. 

